# Intermittent Theta Burst Stimulation Increases Reward Responsiveness in Individuals with Higher Hedonic Capacity

**DOI:** 10.3389/fnhum.2016.00294

**Published:** 2016-06-16

**Authors:** Romain Duprat, Rudi De Raedt, Guo-Rong Wu, Chris Baeken

**Affiliations:** ^1^Department of Psychiatry and Medical Psychology, Ghent UniversityGhent, Belgium; ^2^Department of Psychiatry and Experimental Psychology, Ghent Experimental Psychiatry Lab, University of GhentGhent, Belgium; ^3^Department of Experimental Clinical and Health Psychology, Ghent UniversityGhent, Belgium; ^4^Department of Psychiatry, University HospitalBrussels, Belgium

**Keywords:** iTBS, theta burst stimulation, reward processing, reward sensitivity, anhedonia, dorsolateral prefrontal cortex, probabilistic learning

## Abstract

**Background:** Repetitive transcranial magnetic stimulation over the left dorsolateral prefrontal cortex (DLPFC) has been documented to influence striatal and orbitofrontal dopaminergic activity implicated in reward processing. However, the exact neuropsychological mechanisms of how DLPFC stimulation may affect the reward system and how trait hedonic capacity may interact with the effects remains to be elucidated.

**Objective:** In this sham-controlled study in healthy individuals, we investigated the effects of a single session of neuronavigated intermittent theta burst stimulation (iTBS) on reward responsiveness, as well as the influence of trait hedonic capacity.

**Methods:** We used a randomized crossover single session iTBS design with an interval of 1 week. We assessed reward responsiveness using a rewarded probabilistic learning task and measured individual trait hedonic capacity (the ability to experience pleasure) with the temporal experience of pleasure scale questionnaire.

**Results:** As expected, the participants developed a response bias toward the most rewarded stimulus (rich stimulus). Reaction time and accuracy for the rich stimulus were respectively shorter and higher as compared to the less rewarded stimulus (lean stimulus). Active or sham stimulation did not seem to influence the outcome. However, when taking into account individual trait hedonic capacity, we found an early significant increase in the response bias only after active iTBS. The higher the individual's trait hedonic capacity, the more the response bias toward the rich stimulus increased after the active stimulation.

**Conclusion:** When taking into account trait hedonic capacity, one active iTBS session over the left DLPFC improved reward responsiveness in healthy male participants with higher hedonic capacity. This suggests that individual differences in hedonic capacity may influence the effects of iTBS on the reward system.

## Introduction

Repetitive transcranial magnetic stimulation (rTMS) is a relatively new therapeutic tool to treat major depressive disorder (MDD). Most frequently applied to the left dorsolateral prefrontal cortex (DLPFC), a series of studies have demonstrated its efficiency in the treatment of this disorder (Berlim et al., 2014; Lefaucheur et al., 2014). Studies combining behavioral and neuroimaging data have shown the modulatory effect of high frequency rTMS (HF-rTMS) on different cognitive processes (for a review see Guse et al., 2010). Although the exact working mechanisms of how HF-rTMS treatment improves mood and cognition in MDD patients remains to be elucidated, one possible pathway could be that the mechanisms of action are modulated by the reward system (Downar et al., 2014). It has already been demonstrated in healthy adults that HF-rTMS over the left DLPFC modulates dopamine release in the anterior cingulate cortex, striatum and orbitofrontal cortex and alleviates anhedonic symptoms (Strafella et al., 2001, 2003; Pogarell et al., 2007; Cho and Strafella, 2009).

These regions also play a critical role in reinforcement learning (Santesso et al., 2008; Shohamy et al., 2008; Pizzagalli et al., 2009; Kunisato et al., 2012). Pizzagalli et al. (2008) evaluated the effect of the intake of a single dose of dopamine (D2/D3) agonist (pramipexole dihydrochloride) on reinforcement learning in healthy adults. Participants who received the drug (disrupting dopaminergic neurotransmission) exhibited impaired performance (lower response bias toward the most rewarded stimulus) when compared to placebo. In a similar protocol, Pessiglione et al. (2006) demonstrated that, compared to placebo, the intake of drugs known to enhance dopaminergic neurotransmission (L-DOPA) increased their response bias toward the most rewarded stimulus indicating a sharpened responsiveness to reward. Interestingly, using a non-pharmacological approach, Ahn et al. (2013) assessed the effects of a single HF-rTMS session over the left DLPFC on reward responsiveness in 18 healthy male individuals using a probabilistic reward task (Pizzagalli et al., 2005). After active stimulation only, participants showed significantly increased response bias in the early trials, indicating that a single HF-rTMS session over the left DLPFC increased reward responsiveness toward the most rewarded stimulus. However, as participants were only assessed after the stimulation session and not before, no change to baseline could be examined limiting the interpretations of the results.

Given the importance of examining whether the neurophysiological effects of rTMS are mediated by the reward system, in the current sham-controlled study we wanted to further verify whether in a similar sample of young healthy male individuals, one stimulation session would affect reward responsiveness during probabilistic learning (Pizzagalli et al., 2005). However, individual differences in hedonic capacity, which is the ability to experience pleasure in response to rewarding stimuli, could affect task performance (Sherdell et al., 2012). Therefore, we also assessed the trait hedonic capacity of the participants using the temporal experience of pleasure scale (TEPS) (Gard et al., 2006). As far as we know, the role of individual hedonic capacities on the response to neurostimulation has not yet been investigated in healthy controls.

For the stimulation protocol, we used intermittent theta burst stimulation (iTBS). This kind of stimulation not only reduces significantly the length of the stimulation sessions, making it of high interest for clinical treatment paradigms (Di Lazzaro et al., 2008; Bakker et al., 2015), it is also thought to result in deeper stimulation of the brain and longer lasting stimulatory effects as compared to “classic” HF-rTMS protocols (Huang et al., 2005). We hypothesized that only active iTBS and not sham would positively modulate participants' performance during the completion of the probabilistic learning task. We also hypothesized that trait reward sensitivity would influence the participant's task performance after active iTBS.

## Materials and methods

This study was approved by the local ethics committee of Ghent University Hospital and is in accordance with the declaration of Helsinki (2004). This study was part of a larger project investigating the influence of iTBS on neurocognitive markers in healthy controls and depressed patients.

### Participants

Twenty two healthy male students, all right-handed and naive to TMS, volunteered to participate in this study. Their mean age was 23.2 years (*SD* = 3.59). They had no neurological disorders, psychiatric illness or medical history and were screened by a certified psychiatrist for medical contraindications for rTMS following rTMS safety guidelines (Rossi et al., 2009; Lefaucheur et al., 2014). Participants gave written informed consent prior to the start of the study. Participants were financially compensated for their participation (50 euro + a maximum of 20 extra euros, depending on their performance on the probabilistic learning task).

### Transcranial magnetic stimulation

iTBS stimulation was applied using a Magstim Rapid2 Plus1 magnetic stimulator (Magstim Company Limited, Wales, UK) connected to a 70 mm “figure eight” shaped coil. Before the first stimulation session, the individual resting motor threshold was determined using surface electromyography to measure the minimal stimulation intensity necessary to produce a motor evoked potential on the right abductor pollicis brevis muscle. In order to accurately target the stimulation site [left DLFPC i.e., the center part of the midprefrontal gyrus (Brodmann 9/46)], the Brainsight neuronavigation system (Brainsight™, Rogue Research, Inc.) was used guided by the participant's structural cerebral MRI. After randomization (flipping a coin), participants received one stimulation session (active/sham) using the following parameters: 1620 pulses in 54 cycles of 10 bursts of 3 pulses with a train duration of 2 s and an inter-train interval of 8 s with a power output of 110% of the resting motor threshold. For the sham condition we used a specially designed sham coil identical to the active coil, mimicking the active stimulation feeling and sound without delivering any active stimulation. In this randomized within-subject crossover design, each participant received one active and one sham stimulation session (or vice versa) with an interval of 1 week between the two sessions. For both stimulations, participants were blinded and fitted with ear plugs to limit possible perceptual differences due to the stimulation condition (active or sham). At the start of the experiment, they completed the TEPS questionnaire. Before and after each stimulation, participants were assessed with the probabilistic learning task (Pizzagalli et al., 2005). The order of stimulation was counterbalanced across participants. After exclusion of an outlier, the active-sham group consisted of 10 participants and the sham-active group of 11 participants. On sociodemographic variables (education, marital status, lateralization), the order of stimulation groups only differed in age: active-sham (*M* = 20.80, *SD* = 1.68) sham-active (*M* = 25.45, *SD* = 3.44), *t*_(19)_ = 3.86 *p* < 0.01. This group difference is balanced-out by the crossover design in which each participant acts as his own control.

### Probabilistic learning task (Pizzagalli et al., 2005)

**Figure 1:**
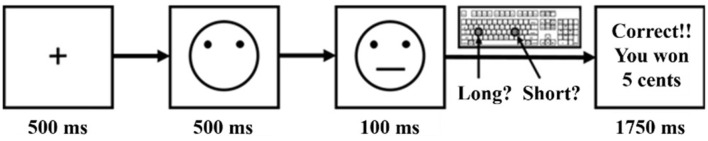
**Probabilistic reward task schematic design (Pizzagalli et al., 2005)**.

The task is composed of three blocks (B1, B2, and B3) of 100 trials. Each trial starts with the presentation of a fixation cross for 500 ms followed by a mouthless cartoon face for 500 ms. A schematic mouth (a horizontal line), long (13 mm) or short (11.5 mm), is then presented on the cartoon face for 100 ms. Participants are forced to choose which stimulus was shown by pressing the corresponding key on their keyboard. The association between a key and a mouth was counterbalanced between participants and between task completion (before vs. after stimulation) to avoid lateralization bias. If not correct, a new trial starts. If correct, participants are sometimes rewarded: a feedback screen announcing that they won 5 eurocents is presented for 1750 ms before starting a new trial (Figure 1). For each block, a pseudo random sequence of 50 short and 50 long mouths is used among which 40 correct responses are programmed to be rewarded. To induce a response bias, one mouth stimulus (called “rich” stimulus) is randomly chosen before the start of the task to be three times more often rewarded when correctly recognized than the other one (called “lean” stimulus). Among the 40 rewarded trials per block, 30 were allocated to the correct recognition of the rich stimulus and 10 to the lean stimulus. The assignment of rich and lean stimuli is counterbalanced within subject across the 4 task completions (if the long mouth is designated to be the rich stimulus for the first task completion, it is automatically designated to be the lean stimulus for the second task completion to avoid repetition during a testing day). Before starting the task, participants are instructed that not all correct trials will be rewarded but they are not informed that one stimulus will be more frequently rewarded than the other one. Participants are instructed to try to win as much money as possible.

### Temporal experience of pleasure scale

The temporal experience of pleasure scale (Gard et al., 2006) is composed of 18 self-report items and assesses individual trait dispositions in both anticipatory (TEPS ANT) and consummatory (TEPS CON) experiences of pleasure (10 items for the anticipatory pleasure scale and 8 items for the consummatory pleasure scale). The sum of the two subscales (TEPS TOT) is a measure of hedonic capacity (or anhedonia; Gard et al., 2006): the lower the score, the lower the hedonic capacity. This scale has the advantage of being applicable in both healthy controls and depressed patients (in depressed patients, low hedonic capacity is referred to as anhedonia), and it has been validated and used as such (Gard et al., 2006; Strauss et al., 2011). It has been demonstrated to have a good internal consistency, test–retest reliability, and convergent and discriminant validity (Gard et al., 2006). As advised by Sherdell et al. (2012) we used this validated scale because of its specificity for hedonic capacity, instead of using separate items from a larger depression scale (i.e., BDI), as they do not provide clear insight into the different subcomponents of reward processing.

### Data reduction and statistical analyses

Three outcome variables were used to assess participant's performance at the probabilistic learning task: response bias (RB), response accuracy and reaction time (RT). The RB is the main dependent variable for this study. It measures the systematic preference of a participant toward the rich stimulus. The RB increases as the participant shows high rates of correct identification for the rich stimulus and low rate of correct identification for the lean stimulus.

$$\begin{array}{l} \text{RB}=\left[\text{log b}=\frac{1}{2}\text{log}\left(\frac{\text{Rich correct }\times \text{ Lean incorrect}}{\text{Rich incorrect }\times \text{ Lean correct}}\right)\right] \end{array}$$

The Response accuracy was also analyzed.

$$\begin{array}{l} \text{Response accuracy }=\;\left(\frac{\text{number of hits}}{\text{number of hits }+\text{ number of misses}}\right) \end{array}$$

Due to low variance in response accuracy, arcsine transformation was performed on raw accuracy data before entering statistical analyses. Regarding reaction time (RT), because the data were not normally distributed, log transformation was also performed before statistical analyses, which resulted in a normal distribution as indexed by the Shapiro-Wilk test and visual inspection of Q-Q plots.

The analyses were performed according to Pizzagalli et al. (2005). Analyses of variance (ANOVA) were performed on transformed accuracy and RT data, with *Condition* (rich, lean), *Stimulation* (active, sham), *Time* (pre, post stimulation) and *Block* (B1, B2, B3) as repeated measures. For response bias, the ANOVA included *Block, Time* and *Stimulation* only. As the aim is to investigate probabilistic learning processes, it is crucial to separate the task in different analytic parts (Blocks) and to include *Block* as a factor in the analysis. Per participant and for each task completion separately, trials with RTs shorter than 3 standard deviations were discarded. For all analyses, the significance level was set at α = 0.05. Cohen's d was calculated to evaluate effect sizes at the contrast level (difference between the means divided by the pooled standard deviation). Where necessary, we applied the Greenhouse-Geisser correction to ensure the assumption of sphericity. All collected data were analyzed with SPSS 22 (Statistical Package for the Social Sciences; IBM SPSS Statistics for Windows, Version 22.0, IBM Corp., Armonk, NY).

## Results

One participant mostly answered using only one key, resulting in either extremely high or extremely low (negative) RB scores with no learning effect throughout the blocks. This indicates that he did not follow the task instructions. The reason for this behavior was not known, as we detected this irregularity only when checking the data later. This participant was consequently removed from the analyses.

### Response bias

In a first step, the repeated measures ANOVA with *Stimulation* (active and sham), *Time* (pre and post stimulation) and *Block* (B1, B2, and B3) as factors showed a main effect of *Block*, *F*_(2, 20)_ = 9.96, *p* < 0.01: the RB in B1 (*M* = 0.04, *SD* = 0.11) was smaller than in B2 (*M* = 0.14, *SD* = 0.17), *t*_(20)_ = 2.67, *p* = 0.01 *d* = 0.70 and the RB in B3 (*M* = 0.20, *SD* = 0.16) was higher than in B1 (*M* = 0.04, *SD* = 0.11), *t*_(20)_ = 4.97, *p* < 0.01 *d* = 1.16. The main effect of *Stimulation* trended toward significance: the RB in the active condition (*M* = 0.15, *SD* = 0.12) was higher than in the sham condition (*M* = 0.10, *SD* = 0.13), *F*_(1, 20)_ = 3.72, *p* = 0.068. No significant *Block* x *Time* x *Stimulation* interaction was found, *F*_(2, 20)_ = 1.88, *p* = 0.16 (For an overview of the ANOVA results, see Table 1; for an overview of the means, see Supplementary Material).

**Table 1:** **ANOVA for the response bias**.

**Variables**	*****df*****	**Mean square**	*****F***-value**	*****P***-value**
Stimulation	1	0.11	3.7	0.068
Time	1	< 0.01	< 0.01	0.99
Block	2	0.55	9.96	< 0.01^*^
Stimulation × time	1	< 0.01	< 0.01	0.95
Stimulation × block	2	0.03	1.01	0.37
Time × block	2	0.15	2.70	0.08
Stimulation × time × block	2	0.10	1.89	0.16

Significant effects are marked by

* p < 0.05.

In a second step, to check the possible influence of individual differences in trait hedonic capacity, the TEPS scores were used as covariates in the analysis. TEPS TOT, TEPS ANT and TEPS CON were entered successively as covariates (ANCOVA). No significant effect emerged from the ANCOVAs using TEPS TOT or TEPS ANT. However, the ANCOVA with TEPS CON as a covariate revealed a significant interaction between *Stimulation, Time, Block* and TEPS CON, *F*_(2, 19)_ = 3.86 *p* = 0.03 (for an overview of the results, see Table 2).

**Table 2:** **Significant or important interactions from the ANCOVA analysis on RB using TEPS TOT, TEPS CON or TEPS ANT as a covariate**.

**Covariate**	**Variables**	*****df*****	**Mean square**	*****F***-value**	*****P***-value**
TEPS TOT	Stimulation × time × block	2	0.08	1.49	0.24
	Stimulation × time × block × TEPS TOT	2	0.09	1.82	0.17
TEPS CON	Time × block	2	0.16	3.16	0.054
	Time × block × TEPS CON	2	0.17	3.47	0.04^*^
	Stimulation × time × block	2	0.14	3.01	0.06
	Stimulation × time × block × TEPS CON	2	0.18	3.86	0.03^*^
TEPS ANT	Stimulation × TEPS ANT	1	0.09	3.57	0.07
	Stimulation × time × block	2	0.05	0.95	0.40
	Stimulation × time × block × TEPS ANT	2	0.05	0.91	0.41

Significant interactions are marked by

* p < 0.05.

Following the significant omnibus interaction with TEPS CON, we ran follow-up tests with TEPS CON as a covariate and check for potential interaction effects with the individual's hedonic capacity (moderation).

At the block level with TEPS CON as a covariate, we looked at the differences between active and sham stimulation at pre- or post-measurements. Only one significant difference was found: B3 pre-active (*M* = 0.23, *SD* = 0.27) was higher than B3 pre-sham (*M* = 0.12, *SD* = 0.26) and this trended toward significance, *F*_(1, 19)_ = 4.26, *p* = 0.053 *d* = 0.41. The interaction also approached significance, *F*_(19)_ = 3.92, *p* = 0.062. For B3 post-active vs. post-sham the interaction with TEPS CON was significant *F*_(19)_ = 6.72, *p* = 0.02.

**Figure 2:**
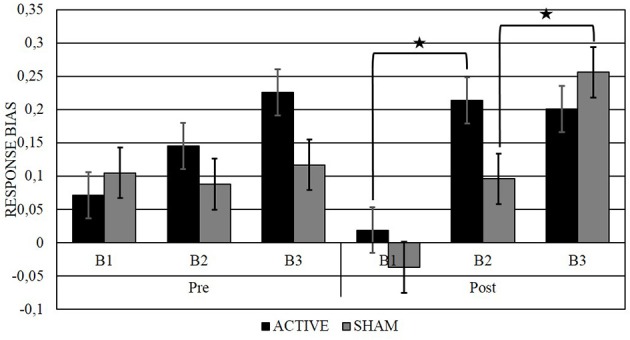
**Representation of the RB across blocks (B1, B2, and B3), before and after (pre, post) active or sham stimulation**. Significant effects are marked by *p < 0.05.

Simple effect analyses with TEPS CON as a covariate were conducted to compare response bias between blocks for each measurement: in the post-active measurement B2 (*M* = 0.21, *SD* = 0.30) was higher than B1 (*M* = 0.02, *SD* = 0.20), *F*_(1, 19)_ = 12.33, *p* < 0.01, with Cohen's *d* indicating a large effect size (*d* = 0.76), and there was no interaction with TEPS CON, *F*_(19)_ = 1.33, *p* = 0.26. In the post-sham measurement B3 (*M* = 0.26, *SD* = 0.32) was higher than B2 (*M* = 0.10, *SD* = 0.35), *F*_(1, 19)_ = 6.00, *p* = 0.02, with Cohen's *d* indicating a medium effect size (*d* = 0.47), and there was no interaction with TEPS CON, *F*_(19)_ = 1.29, *p* = 0.27, (Figure 2). To check whether the order of active vs. sham stimulation would influence the effects, *order* was included as a within-subject factor in a separate ANCOVA in combination with all the other factors. No main effect or crucial interaction with *Order* was found.

For *Time* and *Stimulation*, pre- vs. post-stimulation (active or sham), neither statistical difference nor interaction with TEPS CON was found.

For *Time* and *Block* we looked at the difference between blocks (ΔRB) pre- and post-measurements, and we computed the change post- minus pre-stimulation (ΔRB pre/post) of the differences between blocks for each stimulation.

For ΔRB B2B1, pre-active was not significantly different from post-active, *F*_(19)_ = 2.07, *p* = 0.16, but the interaction with TEPS CON approached significance, *F*_(19)_ = 3.85 *p* = 0.064. Pre-sham vs. post-sham showed no significant difference, *F*_(19)_ = 2.82, *p* = 0.11, and there was no interaction with TEPS CON, *F*_(19)_ = 2.10, *p* = 0.16. Pre-active was not significantly different from pre-sham, *F*_(19)_ = 1.75, *p* = 0.20, but the interaction with TEPS CON approached significance, *F*_(19)_ = 3.71, *p* = 0.07, and post-active vs. post-sham showed no significant difference, *F*_(19)_ = 0.53, *p* = 0.47, and there was no interaction with TEPS CON, *F*_(19)_ = 1.19, *p* = 0.29. For ΔRB B2B1 pre/post-active vs. ΔRB B2B1 pre/post-sham there was no significant difference, *F*_(19)_ = 0.05 *p* = 0.82, and there was no interaction with TEPS CON, *F*_(19)_ = 0.09, *p* = 0.76.

For ΔRB B3B2, pre-active was not significantly different from post-active, *F*_(19)_ = 0.63, *p* = 0.43, but the interaction with TEPS CON was significant, *F*_(19)_ = 7.91, *p* = 0.01. Pre-sham vs. post-sham showed no significant difference, *F*_(19)_ = 2.31 *p* = 0.14, nor interaction with TEPS CON, *F*_(19)_ < 0.01, *p* = 0.95. Pre-active was not significantly different from pre-sham, *F*_(19)_ = 0.52 *p* = 0.48, but the interaction with TEPS CON was significant, *F*_(19)_ = 8.13, *p* = 0.01, and post-active vs. post-sham showed no significant difference, *F*_(19)_ = 2.87 *p* = 0.11, and there was no interaction with TEPS CON, *F*_(19)_ = 1.67, *p* = 0.21. For ΔRB B3B2 pre/post-active vs. ΔRB B3B2 pre/post-sham there was no significant difference, *F*_(19)_ = 2.53, *p* = 0.13, but the interaction with TEPS CON was significant *F*_(19)_ = 19, *p* = 0.03.

For ΔRB B3B1, pre-active was not significantly different from post-active, *F*_(19)_ = 0.09 *p* = 0.76, and there was no interaction with TEPS CON, *F*_(19)_ = 3.24, *p* = 0.09. The pre-sham (*M* = 0.01, *SD* = 0.24) was lower than the post-sham (*M* = 0.29, *SD* = 0.38), *F*_(1, 19)_ = 8.02, *p* = 0.01 *d* = 0.88, but there was no interaction with TEPS CON, *F*_(19)_ = 1.86, *p* = 0.19. Pre-active was not significantly different from pre-sham, *F*_(19)_ = 3.11, *p* = 0.09, and there was no interaction with TEPS CON, *F*_(19)_ = 0.87, *p* = 0.36. Post-active vs. post-sham showed no significant difference, *F*_(19)_ = 1.14 *p* = 0.3, but the interaction with TEPS CON was significant, *F*_(19)_ = 4.86, *p* = 0.04. For ΔRB B3B1 pre/post-active vs. ΔRB B3B1 pre/post-sham there was no significant difference, *F*_(19)_ = 3.42, *p* = 0.08, but the interaction with TEPS CON was significant, *F*_(1, 19)_ = 4.86, *p* = 0.04.

### Correlations

To visualize and further explore possible influential cases related to the nearly significant interaction with TEPS CON and ΔRB B2B1 pre/post active and the significant interaction with TEPS CON and ΔRB B3B2 pre/post active, we ran bivariate correlation analyses on the relationship between TEPS CON and these variables.

**Figure 3:**
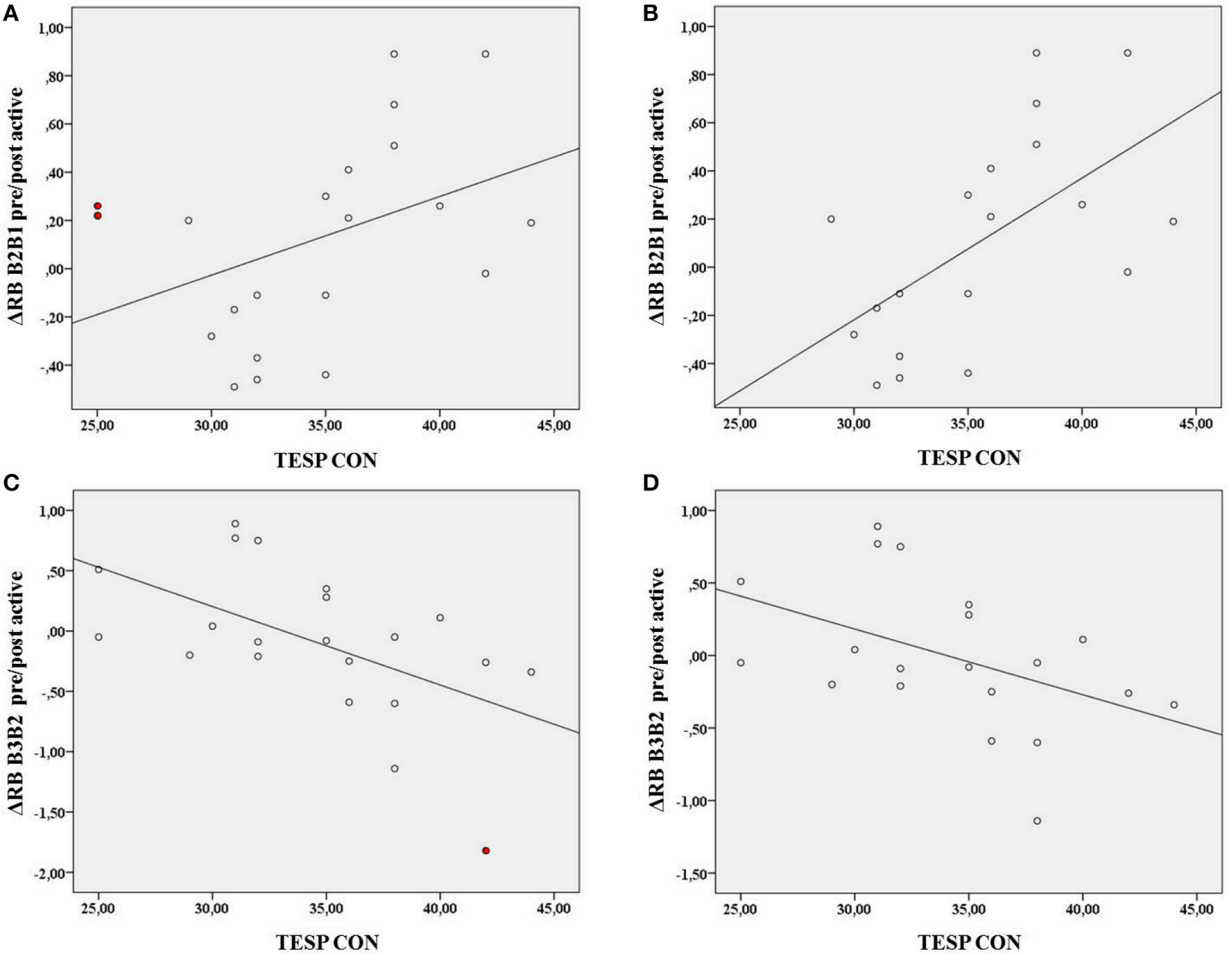
**Correlations between TEPS CON and ΔRB B2B1 pre/post active, *r*_(19)_ = 0.41, *p* = 0.064 (A) and ΔRB B2B1 pre/post active minus the 2 influential cases, *r*_(17)_ = 0.59, *p* < 0.01 (B) and between TEPS CON and ΔRB B3B2 pre/post active *r*_(19)_ = −0.54, *p* = 0.01 (C) and ΔRB B3B2 pre/post active minus 1 influential case, *r*_(18)_ = −0.46, *p* = 0.04 (D)**. The influential cases are colored in red.

For TEPS CON and ΔRB B2B1 pre/post-active (Figure 3A) there was a positive correlation, *r*_(19)_ = 0.41, *p* = 0.064 (see the abovementioned interaction). After visual inspection of the plot of TEPS CON against ΔRB B2B1 pre/post-active, 2 data points appear as influential cases. These 2 cases have the highest scores on both Cook's distance and leverage values (influence measurements) and exceeded the Cook's distance numerical cut-off (Fox, 1991). In addition these 2 data points correspond to the 2 lowest scores in the TEPS CON from our population sample. After removal of these data points, the correlation between TEPS CON and ΔRB B2B1 pre/post-active increased and became highly significant, *r*_(17)_ = 0.59, *p* < 0.01 (Figure 3B).

For TEPS CON and ΔRB B3B2 pre-post-active (Figure 3C) there was a negative correlation, *r*_(19)_ = −0.54 *p* = 0.01 (see the abovementioned interaction). One case exceeded numerical cut-off for Cook's distance but had low leverage value. After removal of this case the correlation decreased but remained significant, *r*_(18)_ = −0.46, *p* = 0.04 (Figure 3D).

### Reaction time

The repeated measures ANOVA with *Condition* (rich and lean), *Stimulation* (active and sham), *Time* (pre- and post-stimulation) and *Block* (B1, B2 and B3) as factors showed a main effect of *Condition, F*_(1, 20)_ = 31.17, *p* < 0.01. Reaction time (log ms) for the rich stimulus (*M* = 6.12, *SD* = 0.16) was faster than for the lean stimulus (*M* = 6.18, *SD* = 0.17). The interaction between *Condition* and *Stimulation* trended toward significance, *F*_(1, 20)_ = 3.49, *p* = 0.076. A significant interaction between *Block* and *Condition* emerged, *F*_(2, 20)_ = 15.89, *p* < 0.01 and the interaction between *Condition, Time* and *Stimulation* approached significance, *F*_(1, 20)_ = 4.25, *p* = 0.052 (for an overview of the ANOVA results, see Table 3; for an overview of the means, see Supplementary Material).

**Table 3:** **ANOVA for the reaction time**.

**Variables**	*****df*****	**Mean square**	*****F***-value**	*****P***-value**
Condition	1	0.44	31.17	< 0.01^*^
Stimulation	1	< 0.01	0.05	0.82
Time	1	< 0.01	0.05	0.82
Block	2	< 0.01	0.12	0.88
Condition × stimulation	1	0.03	3.49	0.08
Condition × time	1	< 0.01	< 0.01	0.97
Stimulation × time	1	0.12	2.25	0.15
Condition × stimulation × time	1	0.02	4.25	0.052
Condition × block	2	0.08	15.89	< 0.01^*^
Stimulation × block	2	< 0.01	0.21	0.81
Condition × stimulation × block	2	< 0.01	0.73	0.49
Time × block	2	< 0.01	1.03	0.37
Condition × time × block	1.45	0.02	2.87	0.09
Stimulation × time × block	2	0.02	1.78	0.18
Condition × stimulation × time × block	2	< 0.01	0.34	0.71

Significant effects are marked by

* p < 0.05.

To investigate the *Condition* x *Stimulation* interaction we compared the RT for the rich and lean stimuli per stimulation condition. In the active stimulation condition, the RT for the rich stimulus (*M* = 6.11, *SD* = 0.19) was faster than for the lean stimulus (*M* = 6.18, *SD* = 0.21), *t*_(20)_ = 4.72, *p* < 0.01 *d* = 0.35. The same results were observed in the sham stimulation condition: RT for the rich stimulus (*M* = 6.13, *SD* = 0.15) was faster than for the lean stimulus (*M* = 6.18, *SD* = 0.15), *t*_(20)_ = 4.38, *p* < 0.01 *d* = 0.26. To specify the significant interaction, we computed the difference of RT between the rich and the lean stimuli for the real stimulation condition (*M* = −0.07, SD = 0.07) and for the sham stimulation condition (*M* = −0.04, *SD* = 0.05). No other contrasts were significant.

Follow-up tests to investigate the *Block* x *Condition* interaction revealed that the average RT for the rich condition decreased along blocks whereas it increased for the lean condition: for the rich condition, RT at B3 (*M =* 6.10, *SD =* 0.15) was significantly faster than at B1 (*M* = 6.14, *SD* = 0.18), *t*_(20)_ = 2.16, *p* = 0.04 *d* = 0.26 whereas for the lean condition, RT at B3 (*M* = 6.19, *SD* = 0.17) was significantly slower than at B1 (*M* = 6.15, *SD* = 0.17), *t*_(20)_ = 2.23, *p* = 0.04 *d* = 0.23.

Follow-up tests on the *Condition* × *Time* × *Stimulation* interaction revealed a significant difference for the rich condition between the post-active and post-sham condition: the average RT was lower in the post-active condition (*M* = 6.09, *SD* = 0.18) than in the post-sham condition (*M* = 6.16, *SD* = 0.16), *t*_(20)_ = 2.5, *p* = 0.02 *d* = 0.38.

We computed the change in RT by subtracting the pre-stimulation to the post-stimulation (ΔRT pre/post stimulation) for each stimulation (active or sham) and stimulus (rich or lean). For the rich stimulus the difference between ΔRT pre/post in the active condition (*M* = −0.04, *SD* = 0.13) and ΔRT pre/post in the sham condition (*M* = 0.05, *SD* = 0.15) tended toward significance, *t*_(20)_ = 1.94, *p* = 0.067 *d* = 0.61. Overall the RT for the rich stimulus decreased after active stimulation whereas it increased after sham stimulation.

We also computed for each stimulus the RT difference in the pre-stimulation condition between active and sham and in the post-stimulation condition between active and sham. For the rich stimulus the comparison of the difference of RT “pre-active minus pre-sham” vs. the difference “post-active minus post-sham” tended toward significance, respectively (*M* = 0.02, *SD* = 0.21) and (*M* = −0.06, *SD* = 0.12), *t*_(20)_ = 1.94, *p* = 0.067 *d* = 0.50 indicating that for the rich stimulus the difference of RT between active and sham was more important after stimulation than before stimulation. When comparing the difference of RT “post-active minus post-sham” of the rich vs. the lean stimulus, the RT difference was more important for the rich (*M* = −0.06, *SD* = 0.12) than for the lean (*M* = −0.01, *SD* = 0.13); *t*_(20)_ = 2.82, *p* = 0.01 *d* = 0.40.

We then compared the differences “active minus sham; post minus pre” values of the rich and lean stimuli to specify how the *Stimulation* and *Time* factors had a different influence on the RT of the two stimuli. The difference between “active minus sham; post minus pre” stimulation was greater for the rich stimulus (*M* = −0.08, *SD* = 0.20) than for the lean stimulus (*M* = −0.04, *SD* = 0.19) indicating that the RT for the rich stimulus was more modulated by the *Stimulation* and *Time* factors than the RT for the lean stimulus but this did not reached significance.

For an overview of the follow-up test means, see supplementary material.

To check for the influence of individual differences in trait hedonic capacity, the TEPS scores were used as covariates in the analysis. TEPS TOT, TEPS ANT and TEPS CON were entered successively as covariates in the abovementioned model (ANCOVA) (see Table 4 for an overview of the results).

**Table 4:** **Significant or important interactions from the ANCOVA analysis on RT using TEPS TOT, TEPS CON or TEPS ANT as a covariate**.

**Covariate**	**Variables**	*****df*****	**Mean square**	*****F***-value**	*****P***-value**
TEPS TOT	Condition × block	2	0.01	3.06	0.06
	Condition × stimulation × time × block	2	< 0.01	0.89	0.42
	Condition × stimulation × time × block × TEPS TOT	2	< 0.01	0.85	0.43
TEPS CON	Condition × block	2	0.03	6.67	< 0.01^*^
	Condition × block × TEPS CON	2	0.02	3.92	0.03^*^
	Condition × stimulation × time × block	2	< 0.01	0.21	0.81
	Condition × stimulation × time × block × TEPS CON	2	< 0.01	0.16	0.85
TEPS ANT	Times × block	2	0.03	3.88	0.03^*^
	Times × block × TEPS ANT	2	0.03	3.95	0.03^*^
	Condition × stimulation × time × block	2	< 0.01	1.05	0.36
	Condition × stimulation × time × block × TEPS ANT	2	< 0.01	1.02	0.37

Significant interactions are marked by

* p < 0.05.

### Response accuracy

A repeated measures ANOVA with *Condition* (rich and lean), *Stimulation* (active and sham), *Time* (pre- and post-stimulation) and *Block* (B1, B2, and B3) as factors was conducted, a main effect of *Condition* emerged, *F*_(1, 20)_ = 24.36, *p* < 0.01: accuracy (arcsine accuracy) for the rich (*M* = 1.14, *SD* = 0.13) stimulus was higher than for the lean stimulus (*M* = 1.02, *SD* = 0.14). Four interactions were also significant: between *Condition* and *Block*, *F*_(2, 20)_ = 11.04, *p* < 0.01; *Time* and *Block*, *F*_(2, 20)_ = 5.74, *p* < 0.01, and between *Condition, Time* and *Block*, *F*_(2, 20)_ = 3.33, *p* < 0.05. The interaction between *Condition* and *Stimulation* approached significance, *F*_(1, 20)_ = 3.97, *p* = 0.06 (for an overview of the ANOVA results, see Table 5).

**Table 5:** **ANOVA for the accuracy**.

**Variables**	*****df*****	**Mean square**	*****F***-value**	*****P***-value**
Condition	1	1.79	24.36	< 0.01^*^
Stimulation	1	0.05	0.80	0.38
Time	1	0.09	2.64	0.12
Block	2	< 0.01	0.29	0.75
Condition × stimulation	1	0.06	3.97	0.06
Condition × time	1	< 0.01	< 0.01	0.99
Stimulation × time	1	0.01	0.39	0.54
Condition × stimulation × time	1	< 0.01	0.06	0.81
Condition × block	2	0.22	11.04	< 0.01^*^
Stimulation × block	2	< 0.01	0.47	0.63
Condition × stimulation × block	2	0.01	0.88	0.42
Time × block	2	0.08	5.74	< 0.01^*^
Condition × time × block	2	0.07	3.33	0.046^*^
Stimulation × time × block	1.56	< 0.01	0.49	0.57
Condition × stimulation × time × block	2	0.03	1.69	0.20

Significant effects are marked by

* p < 0.05.

Follow-up tests on the C*ondition* × *Block* interaction revealed that the average accuracy for the rich condition increased along blocks whereas it decreased for the lean condition. For the rich condition the accuracy at B3 (*M* = 1.18, *SD* = 0.15) was higher than at B1 (*M* = 1.10, *SD* = 0.13) *t*_(20)_ = 2.91, *p* < 0.01 *d* = 0.55; for the lean condition the accuracy at B3 (*M* = 0.99, *SD* = 0.17) was lower than at B1 (*M* = 1.06, *SD* = 0.14), *t*_(20)_ = 4.06, *p* < 0.01 *d* = 0.44. Overall the accuracy for the rich stimulus increased along blocks and between pre and post stimulation whereas for the lean stimulus it remained stable along blocks pre stimulation and decreased post stimulation.

To investigate the interaction between *Time* and *Block,* we compared each block pre- and post-stimulation. The accuracy at B1 was significantly lower in the pre-stimulation condition (*M* = 1.04, *SD* = 0.14) than in the post-stimulation condition (*M* = 1.12, *SD* = 0.13); *t*_(20)_ = 2.99, *p* < 0.01 *d* = 0.52. We also calculated the change in accuracy between blocks for each *Time* condition. Overall, the accuracy increased in the pre-stimulation condition whereas it decreased in the post-stimulation condition. The change between B3 and B1 in the pre-stimulation condition (*M* = 0.05, *SD* = 0.11) was more important than in the post-stimulation condition (*M* = −0.04, *SD* = 0.06); *t*_(20)_ = 3.23, *p* < 0.01 *d* = 1.01. The change between B2 and B1 in the pre-stimulation condition (*M* = 0.02, *SD* = 0.10) was more important than in the post-stimulation condition (*M* = −0.03, *SD* = 0.07) and this difference approaches significance; *t*_(20)_ = 1.97, *p* = 0.063 *d* = 0.58.

Following the *Condition* × *Time* × *Block* interaction, we computed the change in accuracy between block (ΔB2B1, ΔB3B2, and ΔB3B1) for each stimulus pre- and post-stimulation. For the lean stimulus ΔB2B1 pre-stimulation (*M* = 0.01, *SD* = 0.13) was less important than ΔB2B1 post-stimulation (*M* = −0.11, *SD* = 0.11), *t*_(20)_ = 3.47, *p* < 0.01 *d* = 1.00; and ΔB3B1 pre-stimulation (*M* = 0.01, *SD* = 0.14) was less important than ΔB3B1 post-stimulation (*M* = −0.15, *SD* = 0.12); *t*_(20)_ = 3.61, *p* < 0.01 *d* = 1.23. No significant difference was found for the rich stimulus.

We also calculated the change in accuracy between blocks post- minus pre-stimulation (Δ pre/post stimulation) for each condition (rich or lean). The change in accuracy between B2 and B1 pre/post stimulation for the rich stimulus was less important than for the lean stimulus: change for the rich stimulus (*M* = 0.02, *SD* = 0.19) and change for the lean stimulus (*M* = −0.12, *SD* = 0.16); *t*_(20)_ = 2.47, *p* = 0.02 *d* = 0.79. Similarly, the change in accuracy between B3 and B1 pre/post stimulation for the rich stimulus was less important than for the lean stimulus, respectively: (*M* = −0.01, *SD* = 0.16) and (*M* = −0.16, *SD* = 0.20); *t*_(20)_ = 2.48, *p* = 0.02 *d* = 0.80. These results indicate that overall the accuracy for the lean stimulus was more modulated by the *Time* and *Block* factors than the accuracy for the rich stimulus.

Analysis of the effect of *Stimulation* on *Condition* revealed that in the active condition the accuracy for the rich stimulus (*M* = 1.14, *SD* = 0.15) was higher than for the lean stimulus (*M* = 1, *SD* = 0.14); *t*_(20)_ = 5.66, *p* < 0.01 *d* = 0.96. The same effect was found in the sham condition: accuracy for the rich stimulus (*M* = 1.14, *SD* = 0.13) was higher than for the lean (*M* = 1.04, *SD* = 0.16), *t*_(20)_ = 3.48, *p* < 0.01 *d* = 0.68.

For an overview of the follow-up test means, see Supplementary Material.

To check for individual differences in trait hedonic capacity, the TEPS scores were used as covariates in the abovementioned model. TEPS TOT, TEPS ANT and TEPS CON were entered successively as covariates (ANCOVA) (see Table 6 for an overview of the results).

**Table 6:** **Significant or important interactions from the ANCOVA analysis on accuracy using TEPS TOT, TEPS CON or TEPS ANT as a covariate**.

**Covariate**	**Variables**	*****df*****	**Mean square**	*****F***-value**	*****P***-value**
TEPS TOT	Condition × stimulation × time × block	2	0.03	1.76	0.18
	Condition × stimulation × time × block × TEPS TOT	2	0.04	2.00	0.15
TEPS CON	Condition × time × block	2	0.07	3.64	0.04^*^
	Condition × time × block × TEPS CON	2	0.07	3.92	0.03^*^
	Condition × stimulation × time × block	2	0.03	1.55	0.22
	Condition × stimulation × time × block × TEPS CON	2	0.04	2.07	0.14
TEPS ANT	Condition × stimulation × time × block	2	0.03	1.59	0.22
	Condition × stimulation × time × block × TEPS ANT	2	0.03	1.59	0.22

Significant interactions are marked with

* p < 0.05.

## Discussion

The aim of this study was to assess the effects of a single session of iTBS over the left DLPFC on reward responsiveness in healthy male individuals and to check for a possible influence of trait hedonic capacity on the effect of the stimulation. As expected, participants developed a response bias toward the rich stimulus along the task blocks (B1, B2, and B3), indicating that they progressively learned which stimulus was the most often rewarded (Pizzagalli et al., 2005). The RT and response accuracy analysis showed that, as expected, participants were overall quicker to react toward the rich than the lean stimulus and that the accuracy for the rich condition was higher than for the lean condition.

However, this increased reward responsiveness seems to be independent of the type of stimulation (active vs. sham). Although the interaction effects were not significant, in both post-active and post-sham stimulation conditions a significant increase of the response bias was observed and in the post-active stimulation condition the RB increase was observed during the first blocks (B1 and B2) whereas in the post-sham stimulation condition the RB increase was found during the last blocks (B2 and B3). Ahn et al. (2013) reported similar observations: a higher RB during the early trials after HF-rTMS. Surprisingly this increase was limited to the first block of the task and an RB decrease was observed during the second block. Also, no difference in reward learning between blocks was observed. Importantly, and in contrast to our study design, Ahn and coworkers did not perform baseline measurements before the stimulation sessions, limiting the interpretation of these results. As mentioned before, by using a sham controlled cross-overdesign we could not replicate their findings.

However, given our assumption that individual trait reward sensitivity may influence the task performance related to the reward system, participants were assessed before entering the study design with the TEPS. Here our findings showed that only the active stimulation influenced participants' task performance, and that this influence was related to consummatory TEPS scores. Indeed, interactions with the TEPS CON were found in the active stimulation condition (B2B1 and B2B3): a positive correlation between the TEPS CON and the change in the reward learning (pre/post stimulation) between the early blocks (B1 and B2) and a negative correlation between the TEPS CON and the later blocks (B2 and B3) were found. Because the participants developed their RB more importantly during the early blocks, their RB development during the later block decreased and this pattern was correlated with their trait hedonic capacity. The more hedonic the participants the faster they developed their RB after the active stimulation suggesting an increase of their sensitivity to the rewarding stimulus.

This is of interest given that neurostimulation methods can be used to treat depressed patients. For instance, Downar et al. (2014) applied 20 sessions of HF-rTMS on the left DLPFC in 47 MDD patients and compared responders to non-responders. Treatment response appeared to be strongly bimodal showing one group with preserved consummatory hedonic function responding to HF-rTMS and another group with a lower consummatory hedonia ranking (higher consummatory anhedonia) not responding to HF-rTMS. Non-responders also displayed significantly lower connectivity within a classical reward dopaminergic network including the striatum, the caudate nucleus, the ventral tegmental area and the ventromedial prefrontal cortex (vmPC). Within this network the ventromedial prefrontal cortex, which is known for its consistent activation during the experience of rewarding stimulus across studies (Strauss et al., 2011; Diekhof et al., 2012) and thus associated to the consummatory process of reward, was predictive of the treatment outcome.

Interestingly, Vrieze et al. (2013a) found in a study with 79 depressed patients that reduced reward learning as assessed by their performance with the same probabilistic learning task, decreases their odds of remission after 8 weeks of treatment. These observations strengthened the idea of a link between patient's hedonic capability and their response to HF-rTMS. Although our male participants were not clinically depressed, our findings may be indicative of how iTBS treatment may successfully improve mood in one given patient but not the other. Furthermore, in a PET study with 10 healthy volunteers, Vrieze et al. (2013b) demonstrated that dopamine release in the vmPC plays an important role in reinforcement learning.

In our case, the more hedonic the participants (for the consummatory process), the more iTBS could modulate their reward system, increasing dopamine release. Keller et al. (2013), showed in healthy participants that trait hedonia and the functional connectivity within the reward system were positively correlated. Although speculative at this point, higher trait hedonic capacity reflecting stronger functional connectivity between key components of the reward system could explain whether or not cortical stimulation would propagate and modulate deeper structures of the reward system.

In addition, Pizzagalli et al. (2009) showed in a fMRI study that unmedicated depressed patients compared to controls exhibited weaker responses to monetary gains in the left nucleus accumbens and caudate bilaterally but not during reward anticipation, indicating that in depressed patients, the consummatory phase of reward learning might be impaired whereas the anticipatory phase might be preserved. Also in our study no influence of the TEPS anticipatory subscale, in contrast to the TEPS consummatory subscale, was observed. Our results indicate that the more hedonic for the consummatory process the participants were, the more they developed their RB during the early blocks after the active stimulation session. This additive effect being only present in the post-active stimulation and not in the post-sham, it is possible to think that iTBS positively influences reward processing.

The fact that after active iTBS healthy male participants with higher hedonic capacity seem to become more sensitive to reward, makes one wonder whether these neurostimulation parameters could not be contraindicated for patients with bipolar depression. Current rTMS treatment paradigms do not advocate the use of excitatory or high frequency rTMS paradigms in bipolar depression. Indeed, in few cases excitatory stimulation of the left DLPFC has been reported evoking a switch from depression into mania (Lefaucheur et al., 2014). However, only one study to date explicitly examined the effects of HF-rTMS on the reward system, though it was not able to distinguish different clinical effects between uni- and bipolar depression (Downar et al., 2014), leaving this question still open.

Besides the relatively small sample size there are some limitations. First, the interpretations should be limited to young male participants only. Second, even though we used a placebo coil mimicking the physical sensation of the active stimulation, and even though the participants were blinded and used ear plugs during the stimulations sessions, the placebo condition still was not perfect as sound and sensation were different. However, this is a methodological issue affecting almost all sham-controlled rTMS paradigms. Finally, we did not make correction for multiple comparisons. By consequence our results should be interpreted with some caution due to the increased possibility of false positive statistical results.

In conclusion, we could not replicate the results observed by Ahn et al. (2013). However, we found a modulatory effect of trait hedonic capacity on participants' response to iTBS. The higher the hedonic score of the participants, the stronger their reward responsiveness increased after active iTBS. This indicates that individual differences in hedonic capacity may influence the effects of iTBS on the reward system. Neuroimaging studies applying probabilistic paradigms, also in MDD patients, are needed to understand the role of the reward system in the response to neurostimulation treatments.

## Author contributions

All authors contributed to the conception and design, or acquisition of data, or analysis and interpretation of data and drafted the article or revised it critically for important intellectual content and gave final approval of the version to be published. Specifically, RD and CB made substantial contributions to the conception and design of the work; the acquisition, analysis, and interpretation of data for the work; drafted the work and revised it critically for important intellectual content; gave final approval of the version to be published; and agreed to be accountable for all aspects of the work in ensuring that questions related to the accuracy or integrity of any part of the work are appropriately investigated and resolved. RDR and GW made substantial contributions to the analysis and interpretation of data for the work; drafted the work and revised it critically for important intellectual content; gave final approval of the version to be published; and agreed to be accountable for all aspects of the work in ensuring that questions related to the accuracy or integrity of any part of the work are appropriately investigated and resolved.

### Conflict of interest statement

The authors declare that the research was conducted in the absence of any commercial or financial relationships that could be construed as a potential conflict of interest. The reviewer DL and handling Editor declared their shared affiliation, and the handling Editor states that the process nevertheless met the standards of a fair and objective review.

## Abbreviations

rTMS: repetitive transcranial magnetic stimulation
MDD: major depressive disorder
DLPFC: dorsolateral prefrontal cortex
HF-rTMS: high frequency repetitive transcranial magnetic stimulation
TEPS: temporal experience of pleasure scale
iTBS: intermittent theta burst stimulation
TEPS ANT: temporal experience of pleasure scale anticipatory subscale score
TEPS CON: temporal experience of pleasure scale consummatory subscale score
TEPS TOT: temporal experience of pleasure scale total score
RB: response bias
RT: reaction time.
