# Supplementary material for: Intermittent Theta Burst Stimulation Increases Reward Responsiveness in Individuals with Higher Hedonic Capacity
Source: Front Hum Neurosci. 2016 Jun 16;10:294. doi: 10.3389/fnhum.2016.00294 (PMC4910023; doi:10.3389/fnhum.2016.00294)
Supplement: Supplementary file 1 [file Table1.DOCX]

Supplementary Material

Intermittent Theta Burst Stimulation Increases Reward Responsiveness in Individuals with Higher Hedonic Capacity

**Romain Duprat^*^, Rudi De Raedt, Guo-Rong Wu, Chris Baeken**

*** Correspondence:** duprat.romain@gmail.com

| **RESPONSE BIAS** | | | **MEAN** | **S.D** |
| --- | --- | --- | --- | --- |
| **Block** | | | | |
| 1 | | | 0.039 | 0.111 |
| 2 | | | 0.136 | 0.167 |
| 3 | | | 0.200 | 0.162 |
| **Time** | | | | |
| Pre | | | 0.125 | 0.166 |
| Post | | | 0.125 | 0.210 |
| **Stimulation** | | | | |
| Active | | | 0.146 | 0.121 |
| Sham | | | 0.104 | 0.127 |
| **Stimulation x Time** | | | | |
| Active | Pre | | 0.147 | 0.192 |
|  | Post | | 0.145 | 0.198 |
| Sham | Pre | | 0.103 | 0.186 |
|  | Post | | 0.105 | 0.263 |
| **Stimulation x Block** | | | | |
| Active | 1 | | 0.045 | 0.141 |
|  | 2 | | 0.180 | 0.180 |
|  | 3 | | 0.213 | 0.181 |
| Sham | 1 | | 0.034 | 0.160 |
|  | 2 | | 0.092 | 0.193 |
|  | 3 | | 0.186 | 0.183 |
| **Time x Block** | | | | |
| Pre | 1 | | 0.088 | 0.177 |
|  | 2 | | 0.116 | 0.195 |
|  | 3 | | 0.171 | 0.232 |
| Post | 1 | | -0.009 | 0.213 |
|  | 2 | | 0.155 | 0.262 |
|  | 3 | | 0.228 | 0.296 |
| **Stimulation x Time x Block** | | | | |
| Active | Pre | 1 | 0.071 | 0.253 |
|  |  | 2 | 0.145 | 0.247 |
|  |  | 3 | 0.226 | 0.267 |
|  | Post | 1 | 0.019 | 0.197 |
|  |  | 2 | 0.214 | 0.303 |
|  |  | 3 | 0.201 | 0.333 |
| Sham | Pre | 1 | 0.105 | 0.198 |
|  |  | 2 | 0.088 | 0.237 |
|  |  | 3 | 0.117 | 0.265 |
|  | Post | 1 | -0.037 | 0.317 |
|  |  | 2 | 0.096 | 0.353 |
|  |  | 3 | 0.256 | 0.324 |

| **REACTION TIME (Log ms)** | | | | **MEAN** | **S.D** |
| --- | --- | --- | --- | --- | --- |
| **Condition** | | | |  |  |
|  | Rich | | | 6,121 | 0,158 |
|  | Lean | | | 6,180 | 0,167 |
| **Stimulation** | | | |  |  |
|  | Active | | | 6,147 | 0,200 |
|  | Sham | | | 6,154 | 0,148 |
| **Time** | | | |  |  |
|  | Pre | | | 6,147 | 0,171 |
|  | Post | | | 6,153 | 0,172 |
| **Block** | | | |  |  |
|  | B1 | | | 6,149 | 0,174 |
|  | B2 | | | 6,154 | 0,163 |
|  | B3 | | | 6,147 | 0,157 |
| **Condition x Stimulation** | | | |  |  |
| Rich | Active | | | 6,110 | 0,190 |
|  | Sham | | | 6,132 | 0,152 |
| Lean | Active | | | 6,184 | 0,215 |
|  | Sham | | | 6,176 | 0,147 |
| **Condition x Time** | | | |  |  |
| Rich | Pre | | | 6,118 | 0,171 |
|  | Post | | | 6,123 | 0,160 |
| Lean | Pre | | | 6,177 | 0,178 |
|  | Post | | | 6,183 | 0,193 |
| **Condition x Block** | | | |  |  |
| Rich | B1 | | | 6,143 | 0,178 |
|  | B2 | | | 6,119 | 0,159 |
|  | B3 | | | 6,100 | 0,155 |
| Lean | B1 | | | 6,155 | 0,174 |
|  | B2 | | | 6,189 | 0,172 |
|  | B3 | | | 6,195 | 0,167 |
| **Stimulation x Time** | | | |  |  |
| Active | Pre | | | 6,159 | 0,220 |
|  | Post | | | 6,134 | 0,197 |
| Sham | Pre | | | 6,135 | 0,178 |
|  | Post | | | 6,172 | 0,166 |
| **Time x Block** | | | |  |  |
| Pre | B1 | | | 6,147 | 0,212 |
|  | B2 | | | 6,143 | 0,161 |
|  | B3 | | | 6,151 | 0,158 |
| Post | B1 | | | 6,151 | 0,167 |
|  | B2 | | | 6,165 | 0,188 |
|  | B3 | | | 6,144 | 0,172 |
| **Condition x Stimulation x Time** | | | |  |  |
| Rich | Active | Pre | | 6,129 | 0,221 |
|  |  | Post | | 6,091 | 0,180 |
|  | Sham | Pre | | 6,108 | 0,178 |
|  |  | Post | | 6,156 | 0,160 |
| Lean | Active | Pre | | 6,190 | 0,230 |
|  |  | Post | | 6,177 | 0,222 |
|  | Sham | Pre | | 6,163 | 0,186 |
|  |  | Post | | 6,189 | 0,185 |
| **Condition x Stimulation x Block** | | | |  |  |
| Rich | Active | B1 | | 6,134 | 0,221 |
|  |  | B2 | | 6,108 | 0,198 |
|  |  | B3 | | 6,087 | 0,180 |
|  | Sham | B1 | | 6,152 | 0,166 |
|  |  | B2 | | 6,130 | 0,152 |
|  |  | B3 | | 6,113 | 0,174 |
| Lean | Active | B1 | | 6,168 | 0,225 |
|  |  | B2 | | 6,186 | 0,229 |
|  |  | B3 | | 6,197 | 0,217 |
|  | Sham | B1 | | 6,143 | 0,153 |
|  |  | B2 | | 6,193 | 0,153 |
|  |  | B3 | | 6,193 | 0,156 |
| **Condition x Time x Block** | | | |  |  |
| Rich | Pre | B1 | | 6,130 | 0,216 |
|  |  | B2 | | 6,116 | 0,165 |
|  |  | B3 | | 6,107 | 0,161 |
|  | Post | B1 | | 6,156 | 0,171 |
|  |  | B2 | | 6,122 | 0,174 |
|  |  | B3 | | 6,093 | 0,160 |
| Lean | Pre | B1 | | 6,165 | 0,217 |
|  |  | B2 | | 6,170 | 0,172 |
|  |  | B3 | | 6,195 | 0,167 |
|  | Post | B1 | | 6,146 | 0,172 |
|  |  | B2 | | 6,209 | 0,215 |
|  |  | B3 | | 6,195 | 0,204 |
| **Stimulation x Time x Block** | | | |  |  |
| Active | Pre | B1 | | 6,156 | 0,262 |
|  |  | B2 | | 6,147 | 0,237 |
|  |  | B3 | | 6,175 | 0,202 |
|  | Post | B1 | | 6,147 | 0,198 |
|  |  | B2 | | 6,147 | 0,209 |
|  |  | B3 | | 6,110 | 0,207 |
| Sham | Pre | B1 | | 6,139 | 0,232 |
|  |  | B2 | | 6,139 | 0,159 |
|  |  | B3 | | 6,128 | 0,176 |
|  | Post | B1 | | 6,155 | 0,159 |
|  |  | B2 | | 6,183 | 0,187 |
|  |  | B3 | | 6,178 | 0,188 |
| **Condition x Stimulation x Time x Block** | | | | | |
| Rich | Active | Pre | B1 | 6,134 | 0,270 |
|  |  |  | B2 | 6,120 | 0,236 |
|  |  |  | B3 | 6,132 | 0,200 |
|  |  | Post | B1 | 6,135 | 0,202 |
|  |  |  | B2 | 6,096 | 0,197 |
|  |  |  | B3 | 6,043 | 0,184 |
|  | Sham | Pre | B1 | 6,127 | 0,235 |
|  |  |  | B2 | 6,113 | 0,170 |
|  |  |  | B3 | 6,083 | 0,184 |
|  |  | Post | B1 | 6,177 | 0,167 |
|  |  |  | B2 | 6,147 | 0,175 |
|  |  |  | B3 | 6,143 | 0,197 |
| Lean | Active | Pre | B1 | 6,177 | 0,272 |
|  |  |  | B2 | 6,175 | 0,252 |
|  |  |  | B3 | 6,218 | 0,216 |
|  |  | Post | B1 | 6,159 | 0,201 |
|  |  |  | B2 | 6,197 | 0,241 |
|  |  |  | B3 | 6,176 | 0,247 |
|  | Sham | Pre | B1 | 6,152 | 0,234 |
|  |  |  | B2 | 6,166 | 0,168 |
|  |  |  | B3 | 6,172 | 0,184 |
|  |  | Post | B1 | 6,133 | 0,169 |
|  |  |  | B2 | 6,220 | 0,216 |
|  |  |  | B3 | 6,213 | 0,204 |

| **ACCURACY (arcsine)** | | | | **MEAN** | **S.D** |
| --- | --- | --- | --- | --- | --- |
| **Condition** | | | |  |  |
|  | Rich | | | 1,141 | 0,131 |
|  | Lean | | | 1,021 | 0,137 |
| **Stimulation** | | | |  |  |
|  | Active | | | 1,071 | 0,134 |
|  | Sham | | | 1,091 | 0,133 |
| **Time** | | | |  |  |
|  | Pre | | | 1,067 | 0,125 |
|  | Post | | | 1,095 | 0,131 |
| **Block** | | | |  |  |
|  | B1 | | | 1,082 | 0,126 |
|  | B2 | | | 1,075 | 0,122 |
|  | B3 | | | 1,086 | 0,135 |
| **Condition x Stimulation** | | | |  |  |
| Rich | Active | | | 1,141 | 0,150 |
|  | Sham | | | 1,140 | 0,133 |
| Lean | Active | | | 1,000 | 0,140 |
|  | Sham | | | 1,043 | 0,161 |
| **Condition x Time** | | | |  |  |
| Rich | Pre | | | 1,127 | 0,140 |
|  | Post | | | 1,154 | 0,155 |
| Lean | Pre | | | 1,008 | 0,151 |
|  | Post | | | 1,035 | 0,173 |
| **Condition x Block** | | | |  |  |
| Rich | B1 | | | 1,103 | 0,134 |
|  | B2 | | | 1,138 | 0,152 |
|  | B3 | | | 1,181 | 0,147 |
| Lean | B1 | | | 1,060 | 0,140 |
|  | B2 | | | 1,013 | 0,126 |
|  | B3 | | | 0,992 | 0,168 |
| **Stimulation x Time** | | | |  |  |
| Active | Pre | | | 1,052 | 0,158 |
|  | Post | | | 1,089 | 0,126 |
| Sham | Pre | | | 1,082 | 0,136 |
|  | Post | | | 1,100 | 0,150 |
| **Stimulation x Block** | | | | | |
| Active | B1 | | | 1.078 | 0.148 |
|  | B2 | | | 1.066 | 0.132 |
|  | B3 | | | 1.068 | 0.152 |
| Sham | B1 | | | 1,085 | 0.143 |
|  | B2 | | | 1,085 | 0.142 |
|  | B3 | | | 1.010 | 0.144 |
| **Time x Block** | | | |  |  |
| Pre | B1 | | | 1,045 | 0,144 |
|  | B2 | | | 1,065 | 0,137 |
|  | B3 | | | 1,092 | 0,133 |
| Post | B1 | | | 1,119 | 0,132 |
|  | B2 | | | 1,086 | 0,125 |
|  | B3 | | | 1,080 | 0,154 |
| **Condition x Stimulation x Time** | | | |  |  |
| Rich | Active | Pre | | 1,125 | 0,170 |
|  |  | Post | | 1,157 | 0,157 |
|  | Sham | Pre | | 1,129 | 0,151 |
|  |  | Post | | 1,151 | 0,177 |
| Lean | Active | Pre | | 0,980 | 0,190 |
|  |  | Post | | 1,021 | 0,163 |
|  | Sham | Pre | | 1,036 | 0,169 |
|  |  | Post | | 1,050 | 0,206 |
| **Condition x Stimulation x Block** | | | |  |  |
| Rich | Active | B1 | | 1,102 | 0,170 |
|  |  | B2 | | 1,147 | 0,166 |
|  |  | B3 | | 1,174 | 0,165 |
|  | Sham | B1 | | 1,105 | 0,157 |
|  |  | B2 | | 1,128 | 0,154 |
|  |  | B3 | | 1,187 | 0,165 |
| Lean | Active | B1 | | 1,054 | 0,156 |
|  |  | B2 | | 0,984 | 0,137 |
|  |  | B3 | | 0,962 | 0,184 |
|  | Sham | B1 | | 1,066 | 0,159 |
|  |  | B2 | | 1,042 | 0,177 |
|  |  | B3 | | 1,021 | 0,184 |
| **Condition x Time x Block** | | | |  |  |
| Rich | Pre | B1 | | 1,090 | 0,162 |
|  |  | B2 | | 1,116 | 0,178 |
|  |  | B3 | | 1,175 | 0,146 |
|  | Post | B1 | | 1,117 | 0,156 |
|  |  | B2 | | 1,160 | 0,165 |
|  |  | B3 | | 1,186 | 0,193 |
| Lean | Pre | B1 | | 0,999 | 0,167 |
|  |  | B2 | | 1,013 | 0,146 |
|  |  | B3 | | 1,010 | 0,192 |
|  | Post | B1 | | 1,121 | 0,173 |
|  |  | B2 | | 1,012 | 0,169 |
|  |  | B3 | | 0,973 | 0,219 |
| **Stimulation x Time x Block** | | | |  |  |
| Active | Pre | B1 | | 1,044 | 0,177 |
|  |  | B2 | | 1,047 | 0,179 |
|  |  | B3 | | 1,066 | 0,157 |
|  | Post | B1 | | 1,112 | 0,147 |
|  |  | B2 | | 1,085 | 0,111 |
|  |  | B3 | | 1,070 | 0,172 |
| Sham | Pre | B1 | | 1,045 | 0,191 |
|  |  | B2 | | 1,083 | 0,150 |
|  |  | B3 | | 1,119 | 0,147 |
|  | Post | B1 | | 1,125 | 0,143 |
|  |  | B2 | | 1,088 | 0,164 |
|  |  | B3 | | 1,089 | 0,174 |
| **Condition x Stimulation x Time x Block** | | | | | |
| Rich | Active | Pre | B1 | 1,085 | 0,197 |
|  |  |  | B2 | 1,112 | 0,205 |
|  |  |  | B3 | 1,178 | 0,173 |
|  |  | Post | B1 | 1,119 | 0,185 |
|  |  |  | B2 | 1,183 | 0,164 |
|  |  |  | B3 | 1,170 | 0,208 |
|  | Sham | Pre | B1 | 1,096 | 0,213 |
|  |  |  | B2 | 1,120 | 0,198 |
|  |  |  | B3 | 1,172 | 0,178 |
|  |  | Post | B1 | 1,114 | 0,191 |
|  |  |  | B2 | 1,137 | 0,201 |
|  |  |  | B3 | 1,202 | 0,213 |
| Lean | Active | Pre | B1 | 1,003 | 0,225 |
|  |  |  | B2 | 0,981 | 0,217 |
|  |  |  | B3 | 0,954 | 0,219 |
|  |  | Post | B1 | 1,105 | 0,170 |
|  |  |  | B2 | 0,987 | 0,191 |
|  |  |  | B3 | 0,970 | 0,242 |
|  | Sham | Pre | B1 | 0,995 | 0,207 |
|  |  |  | B2 | 1,045 | 0,181 |
|  |  |  | B3 | 1,066 | 0,209 |
|  |  | Post | B1 | 1,136 | 0,200 |
|  |  |  | B2 | 1,038 | 0,242 |
|  |  |  | B3 | 0,975 | 0,245 |
